# Skin aging: are adipocytes the next target?

**DOI:** 10.18632/aging.100999

**Published:** 2016-07-18

**Authors:** Ilja L. Kruglikov, Philipp E. Scherer

**Affiliations:** ^1^ Scientific Department, Wellcomet GmbH, Karlsruhe, Germany; ^2^ Touchstone Diabetes Center, Departments of Internal Medicine and Cell Biology, University of Texas Southwestern Medical Center, Dallas, TX 75390, USA

**Keywords:** skin aging, ultraviolet radiation, dermal adipocytes, adipocyte-myofibroblast transition

## Abstract

Dermal white adipose tissue (dWAT) is increasingly appreciated as a special fat depot. The adipocytes in this depot exert a variety of unique effects on their surrounding cells and can undergo massive phenotypic changes. Significant modulation of dWAT content can be observed both in intrinsically and extrinsically aged skin. Specifically, skin that has been chronically photo-damaged displays a reduction of the dWAT volume, caused by the replacement of adipocytes by fibrotic structures. This is likely to be caused by the recently uncovered process described as “adipocyte-myofibroblast transition” (AMT). In addition, contributions of dermal adipocytes to the skin aging processes are also indirectly supported by spatial correlations between the prevalence of hypertrophic scarring and the appearance of signs of skin aging in different ethnic groups. These observations could elevate dermal adipocytes to prime targets in strategies aimed at counteracting skin aging.

## INTRODUCTION

Skin aging is a continuous process which can be caused by both internal and external factors [1, 2]. It is thought that intrinsic (chronological) and extrinsic (photo-induced) aging are realized through different pathways and can even cause different visual alterations of the skin [3, 4]. These pathways are generally viewed to be connected with modifications of the connective tissue. This is featured in models for classical skin aging that implicate a progressive accumulation of senescent fibrocytes, leading to a subsequent reduction of collagen production and content in the aging skin [5]. Additional scenarios include the excessive generation of superoxides leading to oxidative damage to DNA [6], the local overproduction of matrix metalloproteinases as a result of inflammation or UV irradiation (UVR) [7, 8] and the reduced production of heat shock proteins [9].

Existing theories of skin aging exclude almost completely the involvement of any adipose tissue components. Whereas intrinsic aging is known in general to correlate with a continuous reduction of subcutaneous white adipose tissue (sWAT) and concur-rent accumulation of visceral fat [10], extrinsic aging was till now considered to be fully independent of the state of adipose tissue. The reason for omitting adipose tissue from the equation is related to the low penetration depth of UVR into the human skin. The penetration of light waves into the skin is inversely related to its wavelength [11]. UVR is traditionally subdivided into three classes – UVA (320-400 nm), UVB (280-320 nm) and UVC (100-280 nm). UVA has the highest and the UVC the lowest penetration depth. The light energy transmission through a 70 μm thick epidermis was assessed to be about 0.27% at 290 nm and 9.5% at 313 nm [12]; penetration depths in the forearm skin were measured to be 20 μm at 290 nm and up to 60 μm at 320 nm [13]. Taking into account the characteristic thickness of the facial dermis layer of approximately 1-1.5 mm [14], it is clear that only a fractional part of UVR applied to the skin surface can reach the interface dermis/sWAT and thus directly influence the subcutaneous adipocytes.

These properties of UVR should exclude adipose tissue from the list of global players in extrinsic skin aging. However, based on the results obtained during the recent past, we have reason to believe that adipose tissue should be considered as an important component of the process, in light of the presence and the properties of dermal adipocytes.

### Dermal white adipose tissue – from little-known structure to a global player in skin physiology?

**Figure 1:**
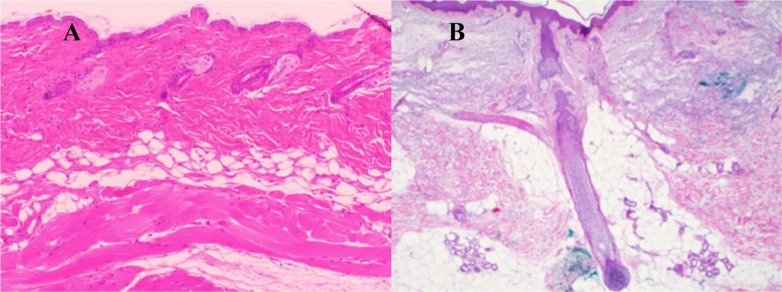
Typical layered dWAT structures in rodents and humans (**A**) Section of the dWAT from the C57/BI6 mouse. This dWAT depot has the layered form placed parallel to the *panniculus carnosus*. (**B**) Human dermal adipocytes in the form of “dermal cones” around the pilosebaceous units. Single “dermal cones” can protrude into the upper dermis. These dermal cones are connected on the other end with sWAT. Pictures courtesy of Drs. Min Kim (**A**) and Travis Vandergriff (4), UT Southwestern Medical Center and published in [32].

Dermal white adipose tissue (dWAT) appears in murine skin as a layer of adipocytes separated from sWAT by the *panniculus carnosus* [15]; in human and porcine skin, it appears in the form of so called “dermal cones” [16, 17], giving raise to the superficial layer of sWAT (Fig. 1). This adipose tissue depot [15, 18] received much recent attention. dWAT is involved in physiologically distinct processes, such as the cycling of hair follicles (HF) [19, 20], wound healing [21], homeostatic temperature regulation of the skin [22, 23], skin protection against infection [24] and cutaneous fibrosis [25]. Moreover, it was hypothesized that dermal adipocytes can be involved in the long-term effects of soft tissue fillers [26], skin hyper-pigmentation, development of hypertrophic scars and at least some skin efflorescences [27, 28]. Important reasons for such unusually multi-functional properties of dermal adipocytes are their high plasticity and the ability to change their phenotype in a very short time [27].

Dermal adipocytes can protrude up to the upper dermis and produce the spatial “fat bridges” between the skin surface and the sWAT (Fig. 1B), thus connecting the regions which can be directly affected by UVR with much deeper fat layers. Paracrine signaling activity of dermal adipocytes discussed in [27] may be responsible for the transduction of the direct response to UVR induced in dWAT to the sWAT layer, providing long-range, deep effects of UVR. At the same time, dWAT has the property to modulate its structure with rates that are much higher than the turnover rates characteristic for adipocytes in sWAT [27]. This can prompt quick structural and functional responses to different external physical insults. These properties provide dWAT the ability to be one of the first-line responders to UVR in the skin.

### Evolution of dWAT during intrinsic aging

Experimental systems to investigate the chronological evolution of damage to the human skin in vivo are very limited. For example, confocal microscopy allows the evaluation of the epidermis and upper dermis; however, alterations in the reticular dermis and in the superficial layer of sWAT cannot be assessed properly with this method, since penetration depth of the laser in the skin is very limited [29]. For this reason we will restrict ourselves mainly on the discussion of aging processes in murine skin where dWAT can be clearly visualized and quantified. To analyze the experimental results concerning the temporal modulation of dWAT, we have o take into account that dWAT demonstrates significant sexual dimorphism [30]. Thus, only animals of the same gender can be compared with each other.

Different experiments revealed an age-dependent evolution of the dWAT thickness in murine skin. One of the most accurate studies of this parameter was provided in [31], where the dWAT thickness was determined in BDF1 female mice for young (6-10 weeks), intermediate (13-19 weeks) and older adult (26-34 weeks) animals. Additionally, these animals were divided into subgroups according to the stage of the hair follicle cycle, since dWAT thickness is known to be dependent on this parameter [19]. This is the reason for the spatially heterogeneous structure of dWAT, even in the same body area [32]. The results reported demonstrate the periodic evolution of dWAT: dWAT is present in 6-week-old mice (anagen phase of HFs), depleted in 9-week-old mice (telogen phase), significantly increased in 12-week-old mice (anagen phase) over its value in 6-week-old animals, reduced in 18-week-old mice (telogen phase) and then strongly increased in adult 31-week-old mice. Interestingly, this dWAT evolution is significantly correlated with skin stiffness, thereby demonstrating the inverse dependence of dWAT on skin thickness, being the lowest in 31-week-old mice. Similar behavior of dWAT was observed in C57BL/6 mice where the dWAT thickness is significantly increased in 12-month-old animals compared to the 2-month-old ones, and this property was found to be sex independent [33]. We also have to consider that the life-span of the C57BL/6 mice, which are often used in these experiments, is on average 29 months in males and 26.5 months in females [34]; thus the evolution between 2 and 12 months can be considered as “maturing”, but not as a “true” aging.

At the same time, age-dependent modifications of dWAT can easily be observed in different knock-out mouse models demonstrating accelerated aging phenotypes. 12-month-old mice carrying a deletion in the cannabinoid 1 receptor gene (Cnr1−/−) demonstrate strongly accelerated aging and have significantly reduced dWAT layers. Such a reduction was observed both in relation to the 2-month-old Cnr1−/− mice and the 12-month-old wild type mice. Another model which is useful in chronological aging studies is the PASG (proliferation associated SNF-2-like gene) null mouse, which also displays distinct signs of premature aging. PASG−/− skin exhibits almost complete depletion of dWAT comparing to the PASG+/+ skin [35]. In addition, p53 mutant mice displaying an early aging-associated phenotype demonstrate depleted dWAT structures in 24-month-old animals (which can be considered as very old), whereas the wild type mice of the same age show significantly reduced but still present dWAT layers [36].

Recapitulating, the chronological evolution of dWAT in intact mice seems to be associated with a periodic modulation of the volume of this depot till mid-age and its subsequent continuous strong involution in old animals. The last feature could be interpreted as a sign of a “true” aging.

### UVR can modulate the metabolism of sWAT

UVR can significantly modulate sWAT metabolism. This effect is observable not only in chronically sun-damaged human skin, but even after a single UV exposure of a non-damaged skin [37]. These authors have shown that the free fatty acid and triglyceride content in sWAT of sun-exposed skin (forearm) is significantly lower than in the buttocks (sun-protected area) of the same subjects. At the same time, young subjects did not demonstrate such differences, which points to the UV-induced effect and not just to the regional variations in fat metabolism. Additionally, both chronic and single UVR exposure significantly reduces master adipogenic factors such as peroxisome proliferator-activated receptor γ (PPARγ); this reduction was rapid and remained stable for at least 72 h after acute UVR exposure. To explain these results, the authors assumed that some soluble factors (such as IL-6, IL-8, MCP-3 and PIGF) produced in upper dermis during UV exposure diffuse into the sWAT and trigger modification of sWAT metabolism. This idea was supported by the fact that the treatment of mature adipocytes from sWAT with these cytokines provides a reduction of triglyceride content. The list of cytokines which could be involved in signal transduction from the skin to the sWAT was further extended in [38], where it was shown that the exposure of preadipocytes to conditioned medium from solar irradiated epidermal-dermal equivalents, containing such inflammatory cytokines as IL-1α, IL-6, IL-11 and TNF-α, inhibited the differentiation of these cells into mature adipocytes. At the same time, application of antibodies neutralizing these cytokines was able to reduce the failure to differentiate significantly. This lead to the conclusion, that inflammatory cytokines are involved in the loss of sWAT during extrinsic aging.

A single low-dose (1.6 J/cm2) of UVB irradiation to Hos:HR-1 hairless mice decreases adiponectin levels in serum and even in peri-ovarian adipose tissue within 24 h after irradiation [39]. This reduction correlated with the observed decreased levels of PPARγ in peri-ovarian adipose tissue. Since the level of serum amyloid A in this study was shown to be significantly increased, the effect of UVB on remote adipose tissue depots was explained through endocrine responses mediated by amyloid A. In addition, reduced obesity was recently demonstrated after chronic exposure of C57BL/6 male mice to UVR independent of vitamin D production [40]. In all experimental studies mentioned above, it was assumed that the targets for UVR are in the epidermis and/or dermis and that the observed reaction of adipose tissue is of an indirect nature. However, since human dWAT structures can spatially reach the upper dermis (Fig. 1B), a direct effect of UVR on the dermal adipocytes cannot be fully excluded. While the lower parts of “dermal cones” (also known as “fat domes”) transverse the dermis and penetrate into the sWAT, the whole reaction chain leading to sWAT modifications can be theoretically realized through adipose tissue directly.

If this model is correct, the rate of extrinsic aging in humans should demonstrate spatial correlations with dWAT structures. Correlations of different pathological processes in the skin with its dWAT content are not unusual and were described, for example, in hypertrophic scarring [16], which predominantly appears in the areas having a high content of “dermal cones”. Analyzing the effects of UVR, these areas must additionally be separated into the sun-exposed and non-exposed areas. The most prominent sun-exposed body areas containing the “dermal cone” structures are the cheek, neck, dorsal hand and forearm; corresponding non-exposed areas containing “dermal cones” are the chest, abdomen, buttock and thigh. Body areas without dermal adipocytes are the palm and scalp [16].

From this point of view dWAT content correlates with a much more pronounced extrinsic aging process in the dorsal hand comparing to the palm area. Chronological skin aging demonstrates similar but not as pronounced differences in aging processes in palmar and dorsal regions of the hand. This can be an indication that UVR accelerates the processes of skin aging, whereas their basic components are determined by some other factors, one of which could be the local dWAT content. This can make skin aging not only body area dependent, but also spatially heterogeneous in the same body area, since dWAT can have a spatially heterogeneous structure [32]. This model allows the appearance of the “mosaic” structure of the aging skin.

### Effects of UVR on the adipocytes in vitro

Irradiation of human adipose-derived stem cells (ADSCs) with UVA in vitro demonstrated suppression of adipogenic differentiation potential of these cells. Such suppression could be observed already by very low fluence of 0.05 J/cm2 and was gradually more severe as fluence increased up to 5.0 J/cm2 [41]. This effect was connected with an observed significant down-regulation of PPARγ expression caused by UVA and demonstrated strong dose- dependent effects. Accumulation of triglycerides in UVA-irradiated cells in this study was also significantly reduced in a dose-dependent manner. Taking into account that UVA of such low fluence as 0.05 J/cm2 was able to induce the pronounced effect on the ADSCs, we can assume that a direct effect of UVR on dermal adipocytes is possible, since such fluences can easily be realized in vivo in the lower dermis. Later, the same group demonstrated that UVA in similar doses also modulates the “stemness” of ADSCs [42].

These results demonstrate that adipocytes can react even to low doses of UVR with a suppression of PPARγ expression and adipogenic differentiation as well as with a reduced accumulation of triglycerides in mature adipocytes. This additionally supports the idea that not only systemic pathways, but also direct local responses in dWAT, can be involved in the reaction of sWAT to UVR in vivo, as observed in [37].

### UVR can cause a reduction of dWAT and trigger cutaneous fibrosis

Chronic (five times weekly, 20 weeks, 16.3 J/cm2 per session) UVA irradiation of hairless mice produced some thickening of the epidermis but no effects in either the upper or lower dermis [43]. However, this irradiation caused the disappearance of dermal adipocytes, the production of fibrosis and a significant increase of hyaluronan content in the lower dermis compared to control animals. Since it was possible to prevent the appearance of cutaneous fibrosis by hydrocortisone, it was concluded that such modifications of the skin structure were caused by an inflammatory reaction induced by UVR.

Similar results were obtained in [44], where histological changes in the skin were investigated in the 11- and 16-week-old female C57BL/6J mice which were chronically (three times weekly, 2-8 weeks, 24 J/cm2) exposed to UVA radiation. Whereas no significant anatomical changes were observed after 2-4 weeks of irradiation, a substantial reduction of dWAT and increased accumulation of collagen fibers were observed after 8 weeks of UVR. The amount of insoluble collagen in the dermis of these mice was found to be approximately 37% higher than in control mice. At the same time, the dermal thickness in 8 week-old UVA-irradiated mice was not statistically different from corresponding age controls which points at the replacement of dWAT layer with cutaneous fibrosis.

**Figure 2:**
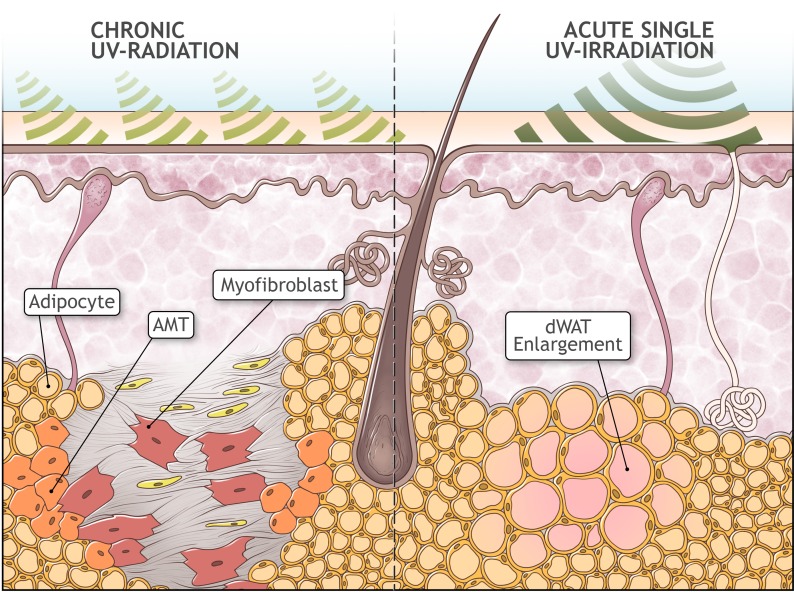
Possible role of adipocyte-myofibroblast transition in extrinsic aging Absorption of UV radiation in the skin causes acute enlargement of the dWAT layer. However, upon chronic overexposure to UV radiation, it causes the depletion of dWAT and a concurrent development of cutaneous fibrosis, presumably through adipocyte-myofibroblast transition (AMT). Replacement of dWAT volume with fibrosis leads to production of mechanically heterogeneous skin structures and to the loss of the effective skin volume.

These earlier results must be re-analyzed, taking into account the recently discovered property of dermal adipocytes to undergo transition into myofibroblast cells (AMT) [25]. This transformation was recognized to be an important pathophysiological step in cutaneous fibrosis. Later it was reported that resistin-like molecule α (RELMα/FIZZ1) can suppress adipocyte-specific genes, triggering a de-differentiation of these cells; at the same time, RELMα/FIZZ1 induced α-smooth muscle actin and type I collagen expression which points to phenotypic transformation of adipocytes into the myofibroblasts [45]. AMT in [25] was induced by subcutaneous injections of bleomycin. AMT has universal features seen under many circumstances, and can be induced by different physical and chemical factors, among them also UVR. From this point of view, the results obtained in [43, 44] can be interpreted as AMT caused by UV exposure (Fig. 2). This effect can be assumed not to be as pronounced as after bleomycin injection. Furthermore, the dWAT cellularity at the time of UVR application should be sufficiently high. This correlates with observations that the replacement of dWAT with cutaneous fibrosis after UVR was observed after sufficiently long UVR exposures as the mice reached the stage of adulthood, which associated with an expanded dWAT depot [31].

Replacement of dWAT with fibrosis through AMT should lead to the production of spatially heterogeneous skin structures. Additionally, this process should prompt the loss of effective skin volume, taking into account the total volume of dermis and dWAT, corresponding to the skin modifications observed in extrinsic aging.

This important suggestion can be directly proven with the help of the recently developed “adipochaser” mouse model [46, 47], which allows to trace the fate of adipocytes in the tissue. If correct, this effect can, at least in extrinsic aging, shift the point of interest from connective tissue to dWAT. We cannot fully exclude that the AMT mechanism is also somehow involved in the intrinsic aging.

### Skin surface lipids and their role in skin reactions to UVR

Skin reactions to UVR are not only dependent on the type and the dose of irradiation, but also on the content of the skin surface lipids (among them prominently α-tocopherol). These lipids are commonly referred to as photo-protectants [48]. The content of these lipids in the skin is significantly reduced after a single UVR application. This can lead to a stronger UV absorption in the skin during a next round of UVR and thus accelerate skin aging. These lipids are believed to be mainly produced and secreted by sebaceous glands which were recognized as the major physiological source for their delivery to the skin surface [49]. At the same time, it was estimated that approximately 90% of the total amount of vitamin E is stored in the lipid droplets of adipocytes and about two-thirds of this content exists in the form of α-tocopherol [50]. Moreover, it is known that α-tocopherol can stimulate the expression of PPARγ and along with that lipid accumulation during adipogenic differentiation [51]. This PPARγ stimulation seems to be realized indirectly through inhibition of its antagonists [52]. Moreover, α-tocopherol can induce the expression of adiponectin, which is in line with its adipogenic effects [53]. These results provide an additional link to dWAT involvement in the skin reaction to UVR.

Adipose tissue displays the slowest turnover for its stored α-tocopherol with an average half-life of 184 days [54]. It is not known whether UVR can speed up the release of α-tocopherol from adipocytes located in dWAT. We would however predict that such an enhanced turnover rate should indeed occur, especially in the case of repeated UVRs that continuously deplete the α-tocopherol pool in the skin and thus force an additional outflow of this lipid from the adipocytes located near the skin surface.

Hence, chronic UVR should deplete the pool of α-tocopherol in dermal adipocytes and thus also suppress the adipogenic differentiation potential and triglyceride accumulation in these cells. This would provide an explanation for the observed continuous reduction of dWAT in the skin chronically irradiated with UV. In contrast, a single UVR may induce a compensatory expansion of dWAT to provide a short-term pool of skin lipids. The reaction of dWAT to UVR should therefore be strongly dependent on the irradiation schedule.

### Immuno-modulators influence adipogenesis and accelerate skin aging

One of the most intriguing phenomena in skin aging is the acceleration of this process through application of different drugs. This effect is known, for example, for immuno-modulators such as cyclosporine A (CsA). Whereas chronic exposure of the Skh-1 hairless mice to non-erythemal doses of UVB induced skin wrinkles after 6-7 weeks of irradiation, concomitant systemic application of CsA reduced the onset time of wrinkling down to 4 weeks [55]. In contrast, HRS/J hairless mice demonstrate no wrinkles after 10 weeks of the same UVB irradiation, and display no wrinkles after 7 weeks of combined application of UVB with CsA.

These results should be re-analyzed taking into account that the dWAT structure and activity in murine skin is dependent on the genetic strain and on the stage of the HF cycle [19]. The main genetic mouse strains used in skin aging experiments are female C57BL/6J mice as well as female Skh-1, HRS/J and albino hairless mice. DWAT in C57BL/6J mice demonstrates a cyclic evolution, which was described in detail in [19]. A mutation of *hr* gene leading to skin baldness causes a disruption of the integrity of HFs as well as the production of utriculi (open comedones) and cysts in the lower dermis and subcutis [56, 57]. Whereas both the Skh-1 [58] and the HRS/J [59] hairless mice have pronounced dWAT layers, HRS/J mice also demonstrate accelerated thymus atrophy [60].

CsA belongs to the group of immuno-suppressors which alter the production of cytokines and influence adipogenesis. This drug can specifically inhibit calcineurin, which is upstream of the nuclear factor of activated T cells (NFAT) transcription factor [61-63]. If NFAT takes part in premature skin aging as observed in [55], dWAT can also be affected, since this pathway is involved in the differentiation of preadipocytes as well [64, 65]. Indeed, chronic application of some immuno-suppressors can reduce both adipocyte size and number [66] and it was proposed that CsA inhibits adipogenic differentiation through prevention of the nuclear localization of NFAT [64]. Hairless mice with thymus atrophy have a marked deficiency in functional T cells, including iNKTs which represent the resident population in adipose tissue [67]. Whereas it is actually not known which level of NFAT expression is typical for dermal adipocytes in athymic nude mice, we assume its expression is altered compared to wild-type animals. This can lead to impaired adipogenic differentiation in the dermis of these animals. This may be one of the reasons for the qualitative differences in effects of CsA on skin aging in different genetic strains observed in [55].

### Sexual dimorphism in dWAT and skin aging

Murine dWAT demonstrates pronounced sexual dimorphism: females have dWAT layers which can be an order of magnitude thicker than in males, whereas the total skin thickness is higher in intact males [30]. After gonadectomy, the dWAT thickness significantly increases both in males and females, whereas treatment of these animals with dihydrotestosterone, 17β-estradiol or dehydroepiandrosterone markedly depletes this depot. Such effects were connected with ability of androgens to inhibit the adipogenic differentiation of stem cells and preadipocytes [68, 69].

A sexually dimorphic response is also known for the response of mice to UVR: males demonstrate a reduced responsiveness to UVA radiation compared to females [70]. Such a response correlates with the lower thickness of dWAT in non-irradiated males and with the ability of UVA to reach the *panniculus carnosus* to affect dermal adipocytes. This again points to the involvement of dermal adipocytes minimally in the context of extrinsic skin aging. Whereas the sexual dimorphism of dWAT in humans was not clearly demonstrated, its gender difference is likely to be present in humans as well. This effect can at least partly explain the difference in the skin aging processes in male and female subjects [71] and will need further intensive investigation.

### Histological evidence for the reaction of dWAT to light irradiation in murine skin

Production of reactive oxygen species (ROS) is one of the primary responses in the skin reaction to UVR [72, 73]. Despite the fact that ROS are often considered to be generally harmful, they in fact able both to stimulate and to suppress the cellular processes, such as the proliferation and differentiation of adipose tissue-derived stem cells (ADSCs) [74]. For example, whereas a high-dose UVB is able to suppress the proliferation of ADSCs, low-dose UVB can increase survival of these cells and up-regulate the expression of different growth factors [75]. Consequently, dWAT and sWAT should react to UVR in a dose-dependent and bi-phasic manner.

The dWAT depot in rodents can demonstrate a quick and significant modulation of its thickness in response to application of different physical factors [27]. In [44], dWAT in C57BL/6J mice was shown to be significantly reduced after UVA irradiation (three times weekly, 8 weeks, 24 J/cm^2^ per session). Similar results were obtained in [76] where the 6-week-old C57BL/6J female mice were exposed to a low-dose UVB radiation (four times weekly, 30 weeks, gradually increasing doses). Histological pictures of the skin in these mice demonstrated a significant reduction of dWAT thickness with a corresponding thickening of dWAT-free dermis layer. Similarly, in [77], dWAT in Skh-1 hairless mice was reduced after UVB irradiation (10 weeks, 3 times weekly, gradually increasing doses from 20 mJ/cm^2^ up to 180 mJ/cm^2^).

At the same time, in [78], the 6-week-old albino hairless mice received a single low-dose UVR (275-380 nm, 200 mJ/cm^2^), which was equal to the minimal erythemal dose for these animals. 72 h after irradiation, skin biopsies clearly demonstrated thickening of the dermis, which was mainly connected with expansion of the dWAT layer. Similarly, UVR (6 weeks, 3 times weekly, minimal erythemal dose) of the 8-week-old HR-1 hairless mice with the wavelengths of 274-380 nm provided significant increase in the thickness and cellularity of dWAT [79]. These results demonstrate a qualitative difference in modification of dWAT structure in C57BL/6J and different models of hairless mice, but they will need to be re-approved taking into account the spatial heterogeneity of dWAT described in [32].

Infrared (IR) radiation with wavelengths up to 1 mm is also able to induce extrinsic skin aging [80, 81]. These light waves have much higher penetration depths than UVR and can thus reach the superficial area of sWAT. From this point of view, it would be interesting to compare the modification of dWAT in IR- and UV-irradiated murine skin. In [82], male Wistar rats were irradiated with IR (1.100-1.800 nm) of 40 J/cm^2^. Skin histology demonstrated abrupt appearance of dermal adipocytes on day 7 after irradiation, with subsequent gradual decrease of their number up to day 180. During the whole observation period, the number of dermal adipocytes was significantly higher in irradiated than in controls. Whereas the action mechanisms of IR and UVR on the skin should to be very different, both types of light seem to have the ability to modify dWAT.

### Additional correlations between UVR and dWAT

#### Possible role of vitamin D modulation in dWAT caused by UVR

Vitamin D can be strongly induced in the skin by UVB radiation [83]. On the other hand, vitamin D is involved in skin aging, since skin aging demonstrates a U-shaped dependence on vitamin D content [84]. It was also shown that vitamin D receptor knockout mice (VDR^−/−^) show a number of signs of premature aging, among them wrinkling skin and a significantly thinner dWAT layer relative to wild type mice [85]. Interestingly, VDR^−/−^ mice demonstrate highly increased expression of the uncoupling protein 1 (UCP1) in sWAT, leading to “beige” fat [86, 87], which correlates with a local loss of sWAT volume. Even more intriguingly, it was shown that these adipocytes express the vitamin D receptor and autonomously generate 1,25-dihydroxyvitamin D_3_ [88], suggesting that adipocytes participate in vitamin D production after UVR.

The dermal adipose layer in murine skin can be depleted by application of 1,25-dihydroxyvitamin D_3_ in high doses; at the same time, this fat depot significantly expands in the absence of vitamin D [89]. This effect can be connected with the ability of 1,25-dihydroxyvitamin D_3_ to inhibit the differentiation of murine preadipocytes through suppression of PPARγ [90]. This reflects the well-known fact that VDR and PPAR signaling pathways are interconnected [91, 92]. Contrarily, both 25-hydroxyvitamin D_3_ and 1,25-dihydroxyvitamin D_3_ can promote differentiation of human preadipocytes [93] and mesenchymal cells [94]. This apparently contradictive influence of vitamin D on the differentiation of murine and human adipocytes still needs to be explained.

Whereas it is actually not known whether dermal adipocytes can produce vitamin D, especially after UVR, it is likely that these cells may indeed do so. In this case, UVB would modulate the dWAT structure also indirectly through induction of vitamin D production in the skin.

#### Possible role of hyaluronan in dWAT modulation

DWAT can be also modulated indirectly through modification of hyaluronan (HA) content in the skin. HA is expressed during adipocyte differentiation; the depletion of HA content was shown to reduce the adipogenic differentiation of preadipocytes *in vitro* as well as the abdominal fat accumulation in C57BL/6J mice [95]. Multiple applications of a hyaluronidase result in significant (up to 35%) reduction of fat mass in the same mouse strain with a simultaneous reduction of the adipocyte size [96]. Not only high-molecular hyaluronan, but also its enzymatically fragmented degradation products inhibit adipocyte differentiation [97]. Many of these findings have been summarized in [98].

UVR can significantly modulate the HA content in different compartments of the skin [99]. Low dose UVB can increase the epidermal synthesis of HA [100], whereas chronic UVB exposure causes loss of HA from the dermis, which is primarily connected with a down-regulation of hyaluronan synthase [101]. Such behavior of HA in the skin correlates with observed expansion of dWAT after low doses of single UVR exposure and depletion of this depot after chronic exposure of the skin to UVR described above.

#### Ethnic differences in skin aging – is dWAT involved?

Whereas the intrinsic aging is believed to occur similarly in different ethnic groups [2], there are well-known ethnic differences in extrinsic skin aging [102-104]. They show higher rates of these processes in Caucasians than in Asians [103, 105], which was generally connected with different melanin content and composition in these ethnic groups [2]. For example, middle-aged Caucasian women demonstrate a much higher appearance of wrinkles than age-matched Asian women [103]. Severe wrinkles on the upper lip were found in 38% and 10% of French and Japanese women aged between 50 and 64 years, respectively. Some types of wrinkles were shown to appear approximately 15 years earlier in French than in Japanese women [103]. Similar differences were observed between German and Japanese women [106]. If dermal adipocytes are involved in the skin aging processes, these differences should be at least partly connected with ethnic variations in dWAT content.

Whereas differences in the skin structure between ethnic groups are well-known [107], direct information about the content of dermal adipocytes in these groups is currently absent. Some further analysis can be done according to the ethnic prevalence of hypertrophic scarring, which correlates with the number of “dermal cone” structures in the skin [16]. Recently, it was shown that the appearance of severe hypertrophic scars indeed significantly varies with race [108], demonstrating 45% higher prevalence among Asians and 78% higher prevalence among African Americans compared with Caucasians. On the other hand, African Americans have bigger sebaceous glands and produce much more lipids than Asians, followed by Caucasians [107]. This correlates with the appearance of hypertrophic scars in these ethnic groups and can at least partly be connected with their dWAT structures.

All this can be an indication for increased content of dWAT in the form of “dermal cones” in the skin of Asians comparing to the same age group of Caucasians, which can, on the other side, be reflected in slower processes of skin aging in the Asian skin.

### Local interaction between fibroblasts and adipocytes

Modification of dWAT caused by extrinsic or intrinsic aging can influence the function of fibroblasts and thus induce the changes in the dermis structure. Enlarged adipocytes significantly suppress the synthetic activity of fibroblasts, whereas small adipocytes do not demonstrate such effects [109]. Very recently, it was also shown that the expansion of sWAT correlates with a decrease of elastic fiber content in the dermis [110]. Consequently, not only chronological or photo-induced aging, but also the local expansion of the adjacent adipose tissue can induce a relatively quick degradation of the elastic fibers in the dermis, which under normal conditions can persist there for many decades [111-113].

## DISCUSSION

During the last few years, it was convincingly demonstrated that dWAT is involved in different physiological and pathological processes in the skin. Remarkably, all observed reactions were connected with significant modulation of the dWAT structure and function. Whereas physiological skin reactions (e.g., immune reaction to pathogens, thermoregulation, etc.) normally cause an expansion of this specialized adipose layer, realized both through hypertrophy and hyperplasia of dermal adipocytes, pathological processes (such as cutaneous fibrosis) were found to correlate with significant dWAT involution.

A number of groups have demonstrated that adipocytes from various superficial and deep fat depots can react to UVR. This interaction can be of direct or indirect nature (e.g., through activation of paracrine signaling or the VDR pathway), depending on the depth of the location of the adipocytes in relation to the penetration depth of UVR. Reduction of triglycerides in sWAT of sun exposed skin and a decrease of adiponectin levels in remote adipose tissue support the indirect mechanism, whereas pronounced histological changes in dWAT after UVR encourage a direct effect of UVR on the adipocytes.

The sexual dimorphism of dWAT and ethnic differences in skin aging provide additional arguments for the involvement of dWAT in the skin aging processes. Female mice have a thicker dWAT layer and demonstrate much higher responsiveness to UVR than males from the same genetic background. On the other hand, ethnic differences in skin aging correlate with the appearance of hypertrophic scars. Taking into account the known spatial correlation between hypertrophic scars and local content of dermal adipocytes, we assume that dWAT should vary in different ethnic backgrounds. This variation may be one of the reasons for the different rates of skin aging in these groups. Verification of this relationship will require a comparison of “dermal cone” structures in populations of different ethnic origin.

Since UVR was shown to cause a depletion of dWAT and the concurrent appearance of dermal fibrosis, one can invoke the recently discovered mechanism of adipocyte-to-myofibroblast transition in the skin reaction to UVR. Whereas this transition in experimental models was mainly induced by sub-cutaneous bleomycin injections, such effects may also be possible as a result of UVR. In this case, dermal adipocytes contribute at least to the extrinsic skin aging processes. This important assumption will need further experimental verification.

The recently proposed involvement of adipogenesis in the long-term effects of the soft-tissue fillers [26] indirectly assumes that the pre-stimulation of such structures can improve the treatment results. In light of all these observations, dermal adipocytes may be an effective target in stand-alone and combinational skin anti-aging therapies.

## CONCLUSION

There are strong indications that the transition of adipocytes to mesenchymal cells substantially contributes to the development of cutaneous fibrosis. It may be an important part of extrinsic skin aging, whereby both the reduction of dWAT and substitution of dWAT volume with fibrotic structures contribute. Since intrinsic (chronological) skin aging is also connected with a progressive reduction of the dWAT layer, it cannot be excluded that an adipocyte-myofibro-blast transition is also involved in this type of skin aging. In the future, this may make dermal adipocytes new interesting targets in anti-aging strategies.

## Abbreviations

ADSC: adipose derived stem cells
AMT: adipocyte-myofibroblast transition
CsA: cyclosporine A
dWAT: dermal white adipose tissue
HA: hyaluronan
HF: hair follicle
IR: infrared radiation
PPARγ: peroxisome proliferator-activated receptor γ
ROS: reactive oxygen species
sWAT: subcutaneous white adipose tissue
UVR: UV radiation
VDR: vitamin D receptor
